# Wideband and high-efficiency spin-locked achromatic meta-device

**DOI:** 10.1515/nanoph-2022-0578

**Published:** 2022-11-24

**Authors:** Xingshuo Cui, Dan Liu, Zanyang Wang, Dengpan Wang, Borui Wu, Guangming Wang, Bin Zheng, Tong Cai

**Affiliations:** Air and Missile Defense College, Air Force Engineering University, Xi’an, 710051, China; State Key Laboratory of Modern Optical Instrumentation, The Electromagnetics Academy Zhejiang University, Hangzhou, 310027, China

**Keywords:** achromatic meta-device, circular polarized, high-efficiency and broadband, metasurface, wavefront manipulation

## Abstract

Achromatic devices present unique capabilities in efficient manipulation of waves and have wide applications in imaging and communication systems. However, the research of achromatic devices is limited by the narrow bandwidth, low efficiency as well as large configurations. In this paper, we propose a general strategy to design spin-locked achromatic metasurface with broadband and high efficiency properties in microwave region. A multi-resonant model is used to control the dispersion within a wide bandwidth by tuning its resonant intensity, resonance numbers as well as resonant frequency. As a proof of the concept, two achromatic meta-devices with ultra-thin profile at microwave frequency are experimentally investigated. The achromatic deflector can reflect the normal incident waves to the same angle within 9.5 to 11.5 GHz, while the other achromatic lens can focus the excitations at the same focal points. The experimentally working efficiency of the meta-devices fluctuates around 71–82% and 57–65% within the target working bandwidth, respectively. Moreover, our meta-devices can preserve the charity of the excitations. The scheme of this research shows great advances in the design of broadband and high-efficiency achromatic devices which can also be applied to other frequency ranges and inspires the realization of ultrabroadband and high-efficiency metadevices.

## 1 Introduction

Chromatic aberration-free meta-device, especially achromatic meta-device plays an essential role in modern science and technology, since it is the basis for almost all wideband communication devices. Conventional achromatic devices are engineered by optimizing the shapes of three-dimensional materials, while they are limited to complex configurations and difficult to integrate. With the advent of metamaterials, the material dispersion can be controlled by tuning the permeability and permittivity. Some interesting phenomena, such as negative refractive index [1–4] and inverse Doppler effect [5] are found and studied. Two-dimensional version of metamaterials, namely metasurfaces, provide more freedom to manipulate the phase, amplitude and polarization [6–8] of the incident electromagnetic (EM) waves. Due to the unique properties, a lot of applications such as ultra-thin meta-lenses [9], beam deflectors [10–12], scattering control [13, 14], multidimensional holograms [15, 16], conversion of propagating waves to surface waves [17], multi-functional polarizers [18–20], and intelligent microwave devices [21, 22] are derived. For a metasurface, the EM characteristics can be controlled by adjusting the propagating phase (the structure size) and/or the geometry phase (rotating the structure) at local positions, and the EM functions can be manipulated by ordering the macroscopic sequence.

This idea provides the basic designing principle for chromatic aberration-free metasurfaces. Guided by the principle, researchers have designed some linearly-polarized achromatic meta-devices in visible and near-infrared wavelengths, such as focusing lenses and beam deflectors [10, 23]. It is a pity that these devices can only work in several separate frequencies [24–27] or at a very narrow band [28, 29]. Due to the arbitrary dispersion control effect, our group has designed wideband achromatic meta-devices [30, 31] and dispersion-enabled cloak [32] with high efficiency in both reflection and transmission geometries.

Despite of the present achievements, there still exists challenges for the circularly-polarized achromatic meta-device. Although previous research has successfully eliminated the chromatic aberration over a continuous wavelength region in the infrared [23] and the visible [33, 34], the working bandwidth expansion is often accompanied with low efficiency. The open issue originating from two aspects. One is that the unit cell could only satisfies the conditions of high-efficiency at certain frequencies, leading to the high-efficiency only at special frequencies for the achromatic meta-devices [23]. Another is that the dispersion control capability is limited for wideband achromatic devices, resulting in the limited efficiency within the whole working band [33, 34]. Therefore, retaining broadband while keeping high-efficiency for achromatic devices under excitation of circularly-polarized waves becomes a thorny problem to be solved urgently.

**Figure 1:**
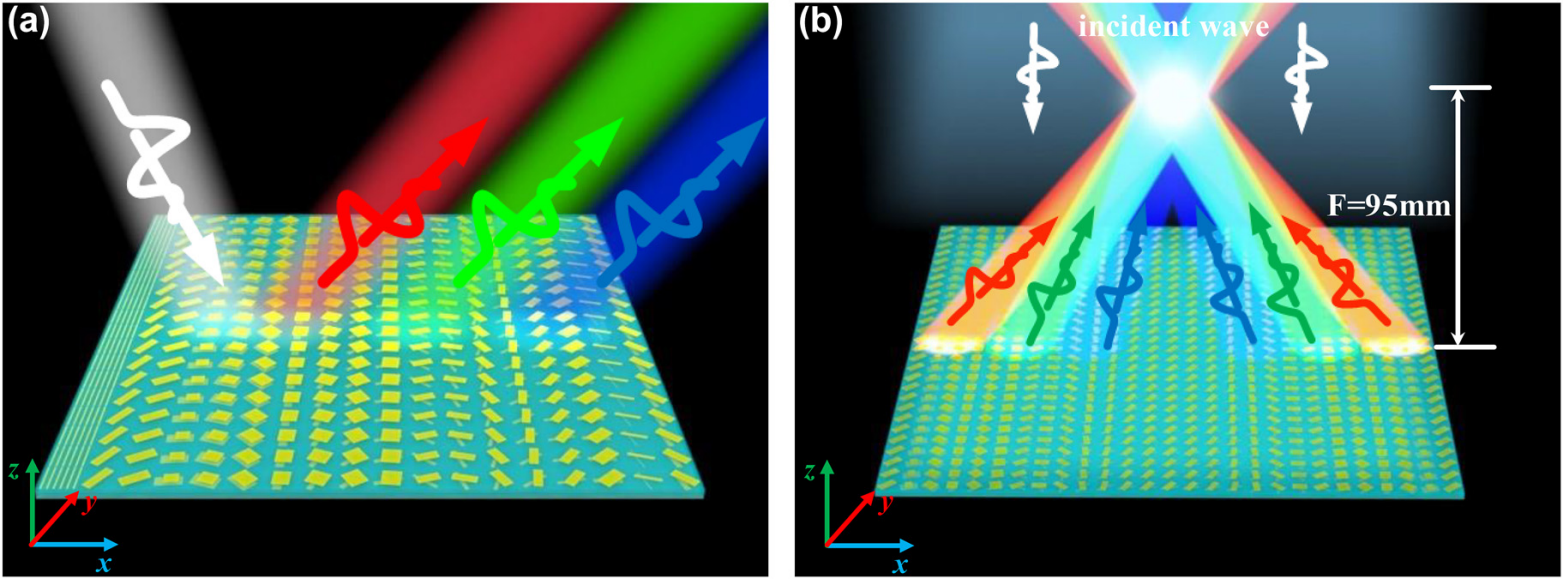
Schematic of the broadband achromatic meta-devices. (a) Beam deflection and (b) achromatic EM waves focusing with the focal length of 95 mm. The RCP plane waves are incident normally on the metasurface and the achromatic performance can be observed at reflective side.

In this paper, we employ the multi-resonant element to control the phase dispersion of circular polarization within wide band by tuning both the propagating phase and geometric phase. The former is realized by optimizing the structure parameters and the latter by rotating the structure. Then, high-efficiency and broadband achromatic meta-devices (beam deflector and focusing metasurface) can be realized in microwave regimes as shown in Figure 1. As a proof of concept, achromatic beam deflector and meta-lens are fabricated and experimented. The reflected EM signal achieves spin-locked property under illustration of right-handed circular polarization (RCP) EM waves. The achromatic devices have stable and brilliant performance in the designed operating bandwidth of 9.5–11.5 GHz. The experimental results show that the highest working efficiency of the achromatic deflector can reach to 82.15% and the achromatic focusing metasurface is 65%. The work innovates designs in controlling circular polarization EM dispersion and push it forward to the practical application.

## 2 Principle and design

For any desired functionality implemented by metasurface, such as beam deflection, focusing and holograms, the critical condition is that the phase shift of the unit cell is required to compensate based on the functionality. The total accumulated phase of the metasurface could be expressed as the sum of two contributions, i.e., the phase produced by the optical path *φ*_*p*_(*x*, *λ*) and the phase generated by the metasurface *φ*_*m*_(*x*, *λ*), as *φ*_tot_(*x*, *λ*) = *φ*_*p*_ (*x*, *λ*) + *φ*_*m*_(*x*, *λ*). *φ*_*p*_(*x*, *λ*) could be expressed as *φ*_*p*_(*x*, λ) = *l*(*x*) × 2π/*λ* where *l*(*x*) is the distance between the interference and the desired wavefront [35, 36]. In principle, we can achieve the desired achromatic beam deflection by tuning the dispersion *φ*_*m*_(*x*, *λ*) as a function of the wavelengths at different position (*x*). Starting with Snell’s generalized reflection law [1, 3], we can derive that *φ*_*m*_(*x*, *λ*) of the achromatic deflector *φ*_*mr*_(*x*, *λ*_*i*_) as:

(1) $$\varphi_{mr}\left(x,\lambda_{i}\right)=-\frac{2\pi }{\lambda }\sin\left(\theta_{r}\right)\left(N_{i}-1\right)p+\varphi_{r0}$$

where the minus sign represents the phase delay and *p* is the size of each unit cell, *N*_*i*_ is the *i*th number of unit cell (*i* = 1, 2…) and *x* depends on the unit cell position and can be dispersed as (*N*_*i*_ − 1)*p*. *φ*_*r*0_ is the initial phase and *θ*_*r*_ is reflection angle when the wave is incident normally on the metasurface.

For the achromatic focusing metasurface, the parabolic phase *φ*_*mf*_ (*x*, *λ*_*i*_) is calculated as:

(2) $$\varphi_{mf}\left(x,\lambda_{i}\right)=-\frac{2\pi }{\lambda }\left(\sqrt{{\left(\left(N_{i}-1\right)p\right)}^{2}+F^{2}}-F\right)+\varphi_{f0}$$

with *φ*_*f*0_ being the initial phase.

It should be mentioned that the initial phase *φ*_*r*0_ and *φ*_*f*0_ are wavelength dependent and they are introduced to optimize the phase compensation effect from the designed deflector and focusing metasurface, which can be defined as follows [23],

(3) $$\varphi_{r\left(f\right)0}=\frac{\alpha }{\lambda }+\beta$$

(4) $$\alpha =\delta \frac{\lambda_{\max}\lambda_{\min}}{\lambda_{\max}-\lambda_{\min}}$$

(5) $$\beta =-\delta \frac{\lambda_{\min}}{\lambda_{\max}-\lambda_{\min}}$$

where *δ* denotes the largest additional phase shift between *λ*_min_ and *λ*_max_ of the initial unit cell of the achromatic deflector or the phase shift at the central position of the achromatic focusing metasurface.

**Figure 2:**
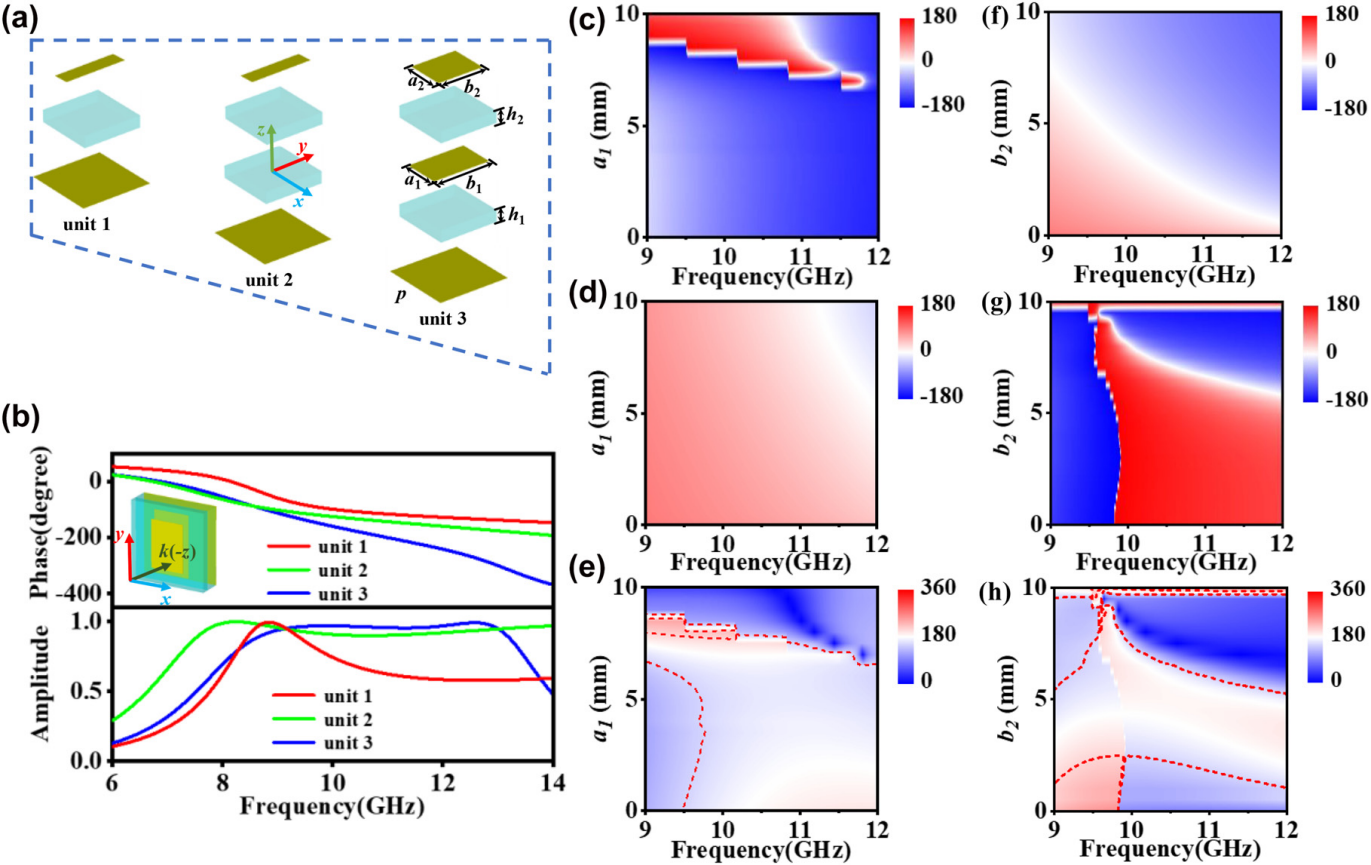
Unit cell configuration and the reflection spectra of the integrated-resonant unit elements. (a) The unit cell elements include different layers of substrate and metal. The substrate is made of F4B with relative permittivity of 2.65, a loss tangent of 0.01*i* and thickness *h*_1_ = 2 mm, *h*_2_ = 1 mm, the period *p* = 10 mm. The metal is made of perfect conductor and the thickness is 0.036 mm. (b) The FDTD simulated reflection amplitude and phase spectra under illustration of RCP EM waves for unit 1 with *a*_1_ = 2 mm, *b*_1_ = 9 mm, unit 2 with *a*_2_ = 2 mm, *b*_2_ = 9 mm, unit 3 with *a*_1_ = 4.6 mm, *b*_1_ = 7.5 mm, *a*_2_ = 4.8 mm, *b*_2_ = 5.9 mm. The phase spectrum of (c) *x*-polarized waves incident, (d) *y*-polarized waves incident and (e) the phase difference various frequency and *a*_1_ when *b*_1_ = 4 mm, *a*_2_ = 8 mm, *b*_2_ = 2 mm. The phase spectrum of (f) *x*-polarized waves incident, (g) *y*-polarized waves incident and (h) the phase difference various frequency and *b*_2_ when *a*_1_ = 4 mm, *a*_2_ = 3.5 mm, *b*_1_ = 8 mm.

The next is to manipulate the dispersion distributions of a reflective unit cell according to the effective medium theory. A sandwich structure, as a typical reflective unit cell which composed of a metallic patch resonator and a continuous metal sheet, separated by a 2 mm thick F4B spacer, is shown in Figure 2(a). The structure can be well described by a Lorentz model, with the reflection phase spectra manipulated by the resonant and plasma frequencies, which is achieved through adjusting the patch structural parameters [31]. The EM behaviors of unit 1 could be calculated through the finite-difference time domain (FDTD) simulation, as shown in Figure 1(b). In the simulation of unit cell, periodic boundary conditions were applied to mimic the infinite size of the structure. It is obvious that there only one resonant frequency at X band and the phase variation range is limited, which couldn’t satisfy the requirement of arbitrary dispersion. This issue could be solved by multi-resonant model which could enlarge the phase variation range and modulate the phase spectra slope [36–38]. Then a double-layered patch structure is introduced as shown in Figure 2(a) as unit 3. The corresponding reflection spectra are calculated in Figure 2(b). It is clear that two resonances of unit 3 are observed clearly. Compared with the spectra of unit 1, the phase variation range is significantly enlarged due to the introduction of another resonance. Therefore, it is the key to realize large phase accumulation by using more resonances. Moreover, we can tune the structure parameters to optimize the phase dispersion. It is interesting that the linear frequency dispersion could be realized by adjusting the resonant frequency and resonant intensity at high-frequency and low-frequency bands, respectively. The underlying physics can be obtained by the current distributions as shown in Figure S1 of Supporting Information. Although the bandwidth can be further extended in our achromatic meta-devices with high efficiency by introducing more resonators, the ratio of the up and low frequencies is limited to ∼2:1. Another structure of unit 2, which the middle layer patch of unit 3 is replaced by vacuum also be considered. The reflective spectra of unit 2 show that there is only a resonance but with more peaceful phase trend compared with the reflective phase of unit 1.

To realize the high efficiency of spin-locked circular polarized metasurface, the phase difference of co-polarized reflectivity for *x*-polarization and *y*-polarization should satisfy *φ*_*xx*_ − *φ*_*yy*_ = π. The EM characters of unit 3 is studied and analyzed when it is illustrated by *x*-polarized and *y*-polarized EM waves respectively. As an example, for the unit cell (*b*_1_ = 4 mm, *a*_2_ = 8 mm, *b*_2_ = 2 mm), the variation of co-polarized reflective phase varying against *a*_1_ and frequencies are shown in Figure 2(c) (*x*-polarized) and Figure 2(d) (*y*-polarized). The phase difference *φ*_*xx*_ − *φ*_*yy*_, shown in Figure 2(e), indicates that phase difference could maintain around 180° among 9.5–11.5 GHz when *a*_1_ is adjusted up to 8 mm and the phase difference between 180° ± 30° is outlined by red dotted line. It is clearly that the two-layered patch structure could achieve high-efficiency spin-locked reflection when manipulating the phase dispersion. The co-polarized reflective phase spectrum of another unit cell (*a*_1_ = 1.5 mm, *b*_1_ = 8.5 mm, *a*_2_ = 6 mm) is shown in Figure 2(f) (*x*-polarized) and Figure 2(g) (*y*-polarized). The variation of phase difference with frequencies (Figure 2(h)) also verifies that the unit cell could maintain high-efficiency spin-locked reflection when *b*_2_ changes from 2.4 to 5.2 mm. The variation of reflective phase spectrum with other parameters also studied and the details are revealed in Figure S3 of Supporting Information.

Further, when the orientation of top and middle metallic patches of unit 3 is tuned together, it matches with the Pancharatnam–Berry (PB) principle [39]. Thus, the variation of the phase is twice as the rotation angle of the patches. This provides the new freedom to adjust the phase. The details are shown in Figure S4(a) of Supporting Information. The combination of propagating phase (the structure size) and the geometry phase (rotating the structure) can realize the required dispersion for achromatic meta-devices with high efficiency and wideband properties.

## 3 Meta-device implementation

### 3.1 Achromatic beam deflector

**Figure 3:**
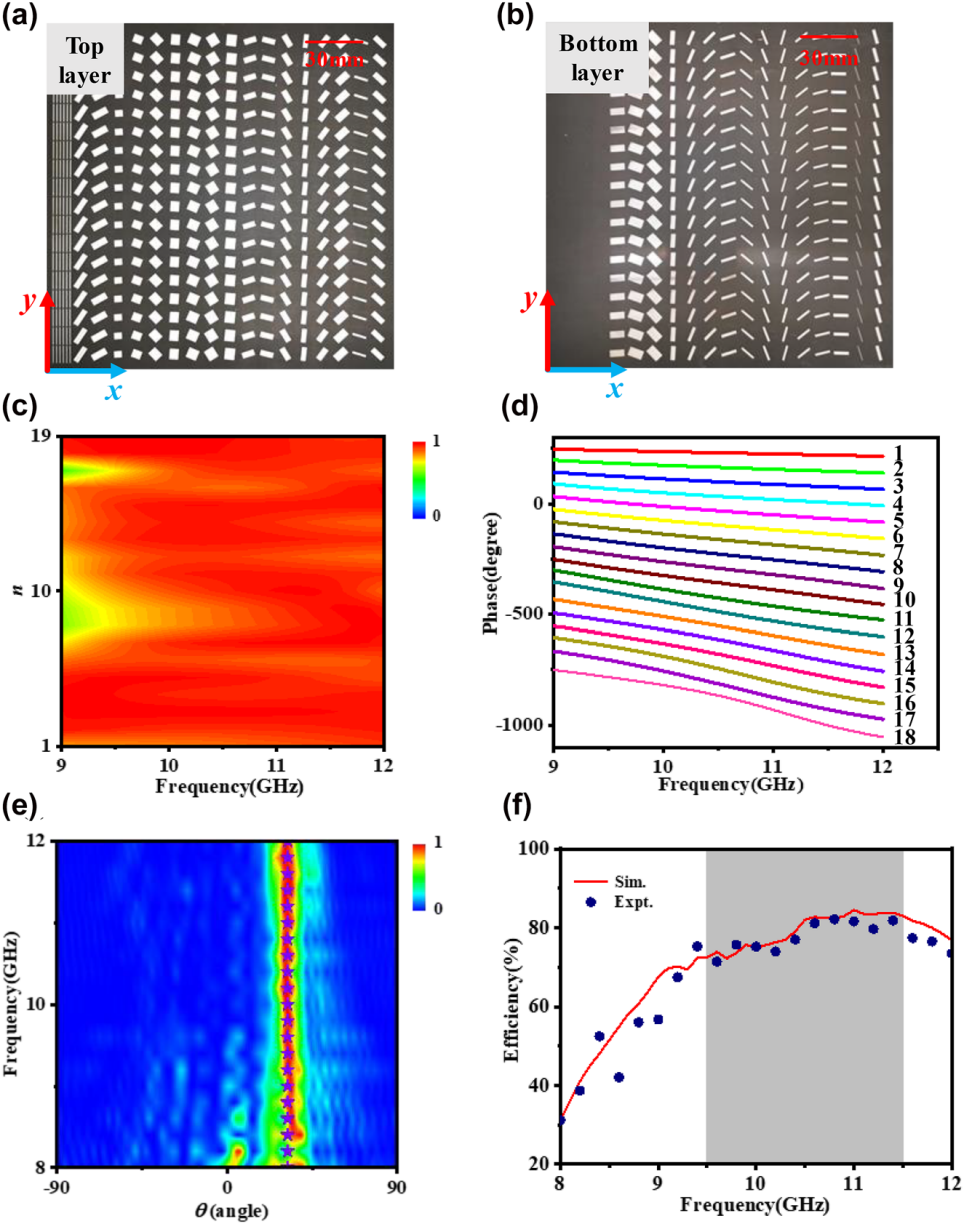
Design of achromatic beam deflector. (a) Top layer and (b) bottom layer of the fabricated achromatic beam deflector. (c) The amplitude spectrum and (d) phase profile of each unit cell from FDTD simulation. (e) Experimental far-field patterns against frequency and detection angles within the operation band. The modena solid star in (e) represents the theoretical value of deflection angle. (f) Simulation and experimental efficiency of the achromatic beam deflector.

To verifying the idea, an achromatic beam deflector working from 9.5 to 11.5 GHz is designed as its schematic shown in Figure 1(a). Here, the deflection angle at working band is set as 32° while the total number of unit cells is chosen as 18. The phase changes in the *x* axis direction based on Eq. (1) and remains constant in the *y* direction. In addition, the first and second elements are derived from the type of unit 2 while the other unit cells are composed of two-layered patch structures as type of 3. The designed phase responses against frequency are shown in Figure 3(d), with the amplitude distributions in Figure 3(c). It obvious that the amplitudes of the reflection coefficient remain at high levels better than |*t*| > 0.83 while the phases change linearly with different slopes for the designed unit cells. The specific values of parameters are shown in Figure S5 of Supporting Information.

After optimization and simulation, a sample of the achromatic deflector is fabricated using the standard printed circuit board technology. Top view of the fabricated sample is shown in Figure 3(a) (top layer) and Figure 3(b) (bottom layer) and the total size is 180 mm × 180 mm (18 × 18 unit cells) with a total thickness of 3.1 mm (0.12*λ*_min_). The experiment is carried out in an anechoic chamber. In our experiment, the sample is illuminated normally by RCP EM waves from an RCP horn antenna and the scattered wave is received by another RCP horn antenna at the reflection side of the metasurface, as shown in Figure S10. The two horn antennas are connected to a vector network analyzer (Agilent E8362C PNA) by coaxial cables. As Figure 3(e) shows, most of the incident waves are bended to the same angle of 32° within 9.5–11.5 GHz, which is consistent with the theoretical calculation, demonstrating the achromatic deflection property. Moreover, most of the undesired modes of scattering waves are suppressed. Thus, the performance ensures the high working efficiency. Outside the target bandwidth, undesired modes increase significantly. The simulations can duplicate the experimental results which are shown in Figure S6 of Supporting Information.

Further, we make quantitative analysis of the measurement results and calculate the working efficiency. The working efficiency is defined as the ratio between the power of expected deflection mode and the incident power at each frequency [11, 31, 40]. The power of the desired mode is obtained by integrating the main lobe of anomalous reflection at different frequencies and the incident power is calculated by integrating the power of reflection mode when the metasurface is replaced by a metallic plate with the same size. The simulation and experimental working efficiency are shown in Figure 3(f). At the target bandwidth 9.5–11.5 GHz, the absolute efficiency of the deflector is with the range of 71–83% retrieved from the FDTD simulation results and it is 71–82% based on the experiment results. The missing power is caused by the dielectric loss and other undesirable reflection.

### 3.2 Achromatic focusing metasurface

**Figure 4:**
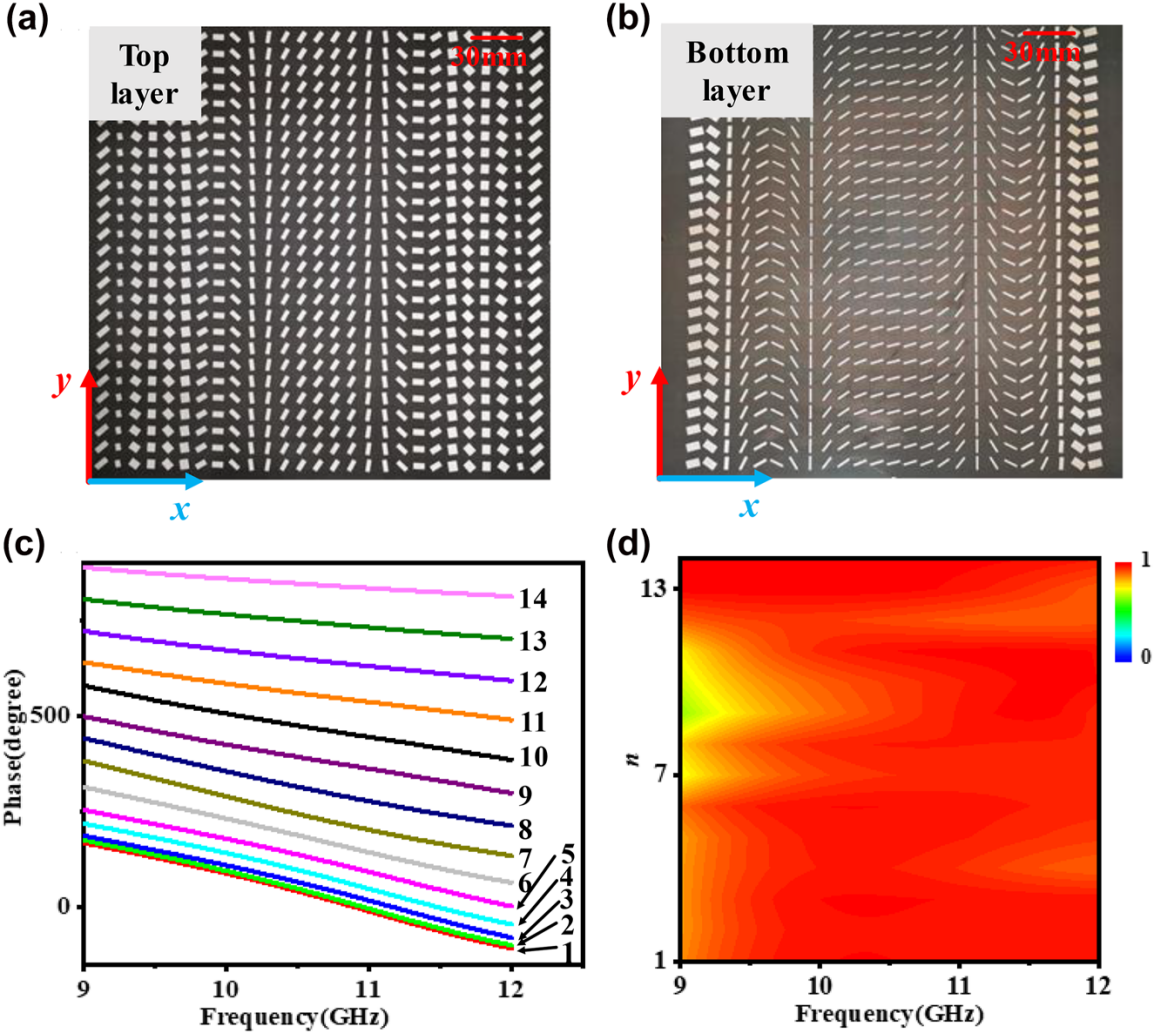
Design of the achromatic meta-lens. (a) Top layer and (b) bottom layer of the fabricated sample of achromatic meta-lens. (c) The phase profile and (d) amplitude spectrum of the achromatic meta-lens.

A reflective circular polarization achromatic focusing metasurface working within 9.5–11.5 GHz is designed by using the previously explored unit cell structure. The metasurface is set as the unidimensional focus as shown in Figure 1(b) while the focal length is set as 95 mm. At first, we calculated the phase distribution of the metasurface based on Eq. (2), as shown in Figure S7(a) of Supporting Information. The phase has a parabolic distribution in *x* axis direction and is constant in *y* axis direction. The unit cells of the metasurface along *x* axis are symmetrically distributed with respect to the center units. The phase spectra of all optimized 14 unit cells are shown in Figure 4(c) and the amplitude spectra of them are shown in Figure 4(d). We find that the reflectivity keeps high level according to the spectrum of amplitude, which guarantees high efficiency of the achromatic focusing metasurface. Correspondingly, the specific value of the parameters is displayed in Figure S7(b) of Supporting Information.

**Figure 5:**
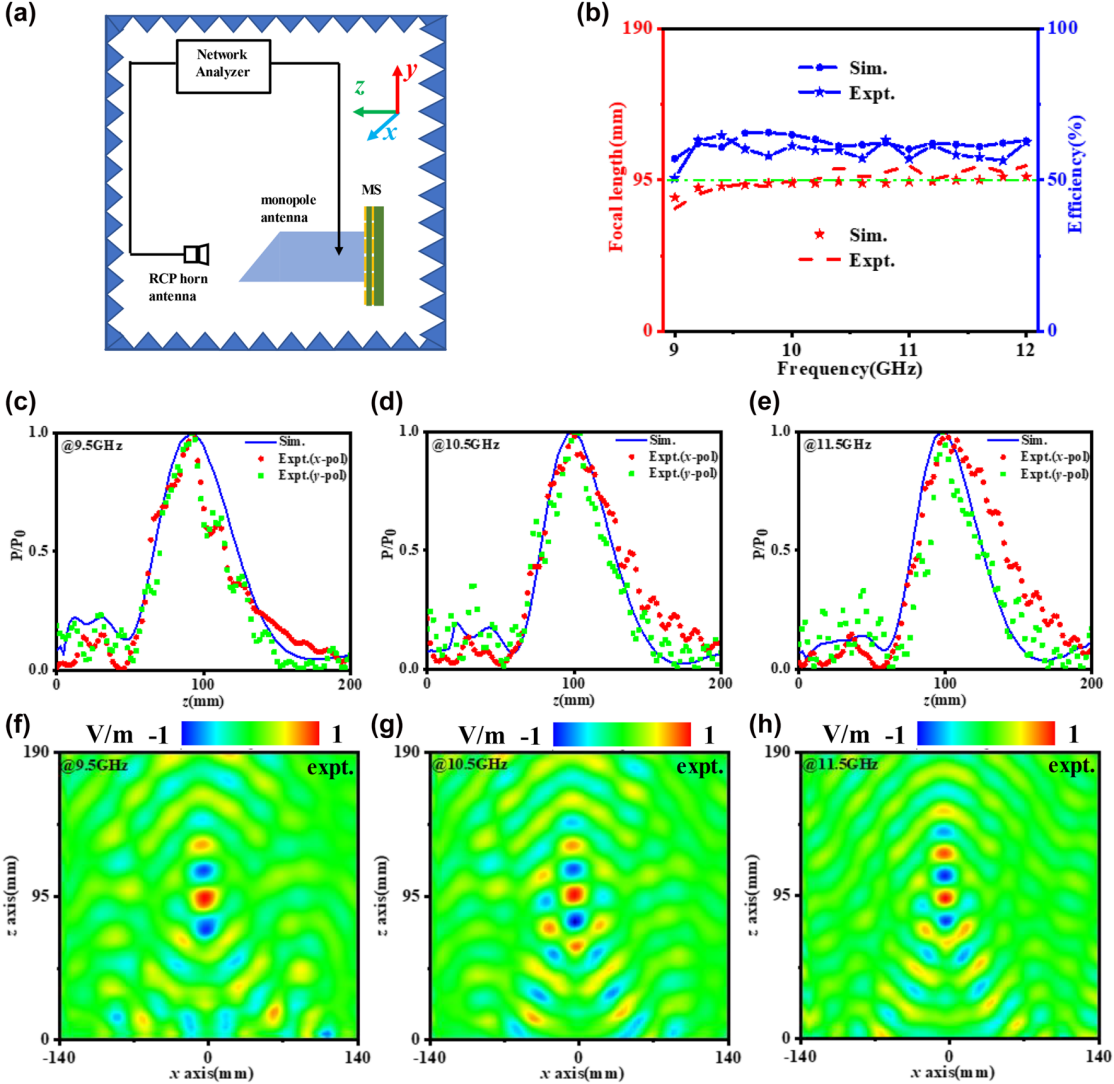
The experimental set ups and results. (a) Schematic of the near field experimental environment. (b) The simulation and experiment working efficiency and focal length varying against frequency. Simulation and experimental normalized energy distribution along *z*-axis at (c) 9.5, (d) 10.5, and (e) 11.5 GHz. Experiment results of near-field distribution for *x*-polarization at (f) 9.5, (g) 10.5, (h) 11.5 GHz.

To further validate the design, we fabricated an achromatic meta-lens which contains 27 × 27 unit cells and occupies a total area of 270 mm × 270 mm. Figure 4(a) and (b) show the top view of the top substrate and bottom substrate. Then, we conduct an experiment of the sample to characterize the metasurface. The schematic of experiment setup is shown in Figure 5(a) and more details are shown in Figure S10 of Supporting Information. The origin of Cartesian coordinate system is set in the center of metasurface. The metasurface is shined by an RCP horn antenna and a monopole antenna is used to detect the scattering EM waves, all of them are connected to a network analyzer. The monopole antenna is placed along *xoy* plane, as shown in Figure 5(a), to collect electric field of *x*-polarized component and *y*-polarized component, including amplitude and phase information. The normalized energy distribution (|*E*|^2^) along *z*-axis is displayed in Figure 5(c)–(e). For accuracy, the experimental results are extracted from *x*-polarized and *y*-polarized electric field component severally. The experimental results are in good agreements with simulation results. It is not difficult to find that the peak of energy both appear around 95 mm at three respective frequencies of 9.5, 10.5, 11.5 GHz. The results prove the accuracy of the unit cell phase design. The differences between simulation and experiment results may have originated from imperfect incident wave front from the transmitting horn. The sample fabrication error and noise error also effect the accuracy.

Then the experimental results are quantitatively analyzed. At first, the achromatic property is considered. Focal length equals to the vertical dimension between the position of all reflective energy peak and metasurface. The simulation focal length (marked with red pentacle) and experimental focal length (marked with red dotted line) varying against frequency is shown in Figure 5(b). It is not difficult to find that the focal length within the band 9.5–11.5 GHz is around 95 mm and the fluctuation range is within 3 mm. The efficiency of the sample is calculated with the product of reflecting efficiency and focusing efficiency. The reflecting efficiency *η*_1_ is defined as the ratio between the power taken by the reflective EM of metasurface and the total incident power at different frequencies. A metal plate with the same size of the metasurface is used as a reference. The total reflected waves are received, integrated and defined as *P*_tot_. And then, the reference metal plane is replaced by the metasurface and the spin-locked reflected waves are received. The main lobe is integrated, defined as *P*_ref1_. The focusing efficiency *η*_2_, defined by the ratio of the focusing power and the reflecting power at central focusing section *xoz*. The integration of reflective power along the *x*-axis direction which containing the focus on central focusing section (*xoz* plane, *y* = 0 mm) is defined as reflecting power *P*_ref2_ while the integration of power concentration interval is defined as focusing power *P*_foc_. More detailed calculation is explained in Section F of Supporting Information. Thus, the working efficiency *η* could be calculated in the formula as:

(6) $$\eta =\eta_{1}\times \eta_{2}=\frac{P_{\text{ref}1}}{P_{\text{tot}}}\times \frac{P_{\text{foc}}}{P_{\text{ref}2}}$$

The efficiency of the metasurface is in the range of 61–66% for the simulation results and 57–65% for the experimental results, as shown in Figure 5(b). It manifests that the achromatic metasurface could working in high efficiency and the performance is stable. Also, the electric field maps of *xoz* plane (*y* = 0 mm) at 9.5, 10.5, 11.5 GHz are taken in Figure 5(f)–(h), which shows that the *x*-polarized electric field component of EM waves go through metasurface and the achromatic focusing effect is obvious. For the *y*-polarized electric field component of EM waves, the fine focusing effect could be seen from Figure S9 of Supporting Information.

## 4 Conclusions

To sum up, a general strategy to design high-efficiency and broadband spin-locked achromatic devices via exploiting geometric phase (PB phase) combining with propagation phase is proposed. For demonstration, two broadband achromatic metasurface devices (achromatic beam deflector and achromatic meta-lens), which can suppress the chromatic aberration in a continuous bandwidth, are designed and fabricated. The operating bandwidth of the devices is 9.5–11.5 GHz (19%) under incidence of the RCP waves and the reflected EM waves keep the same charity. For the achromatic beam deflector, anomalous reflection of 32° can be observed with high-efficiency (71–82%) and wideband performance within the spectra. The achromatic meta-lens possesses the ability to focus the reflected EM waves with the focal length of 95 mm with efficiency in the range of 57–65%. The exploration of retaining achromatic properties in a broad band range paves a way to advance the development of spin-locked metasurfaces.

## Supplementary Material

Supplementary Material Details
