# Supplementary material for: Wideband and high-efficiency spin-locked achromatic meta-device
Source: Nanophotonics. 2022 Nov 24;12(1):119–27. doi: 10.1515/nanoph-2022-0578 (PMC11501814; doi:10.1515/nanoph-2022-0578)
Supplement: Supplementary file 1 — Supplementary Material Details [file j_nanoph-2022-0578_suppl.docx]

**Supporting Information**

**Wideband and high-efficiency spin-locked achromatic meta-device**

Xingshuo Cui^1, 3^, Dan Liu^2, 3^, Zanyang Wang^1, 3^, Dengpan Wang^1^, Borui Wu^1^, Guangming Wang^1, *^, Bin Zheng^2, *^, and Tong Cai^1, 2, *^

*^1^Air and Missile Defense College, Air Force Engineering University, Xi’an, 710051, China*

*^2^State Key Laboratory of Modern Optical Instrumentation, the Electromagnetics Academy Zhejiang University, Hangzhou, 310027, China*

*^3^These authors contributed equally*

**Corresponding Authors: caitong326@sina.cn (Tong Cai), zhengbin@zju.edu.cn (Bin Zheng), wgming01@sina.com (Guangming Wang)*

A. The underlying physics of the two-layered unit structure........….............................2

B. Principle of phase control for the unit cell of meta-devices......................................3

C. Detailed design of achromatic deflector..........…......................................................6

D. Simulation results of scattered-field patterns for achromatic deflector….…………7

E. Detailed design of achromatic focusing metasurface................................................7

F. The performance of achromatic focusing metasurface..............................................8

G. Measurement setup for the designed meta-devices.................................................10

1. **The underlying physics of the two-layered unit structure**

The proposed meta-atom can achieve reflection phase manipulation within a wide frequency band of 9.5-12 GHz. In this section, we illustrate the underlying physics via analyzing the current distribution characteristics of the unit-cell.


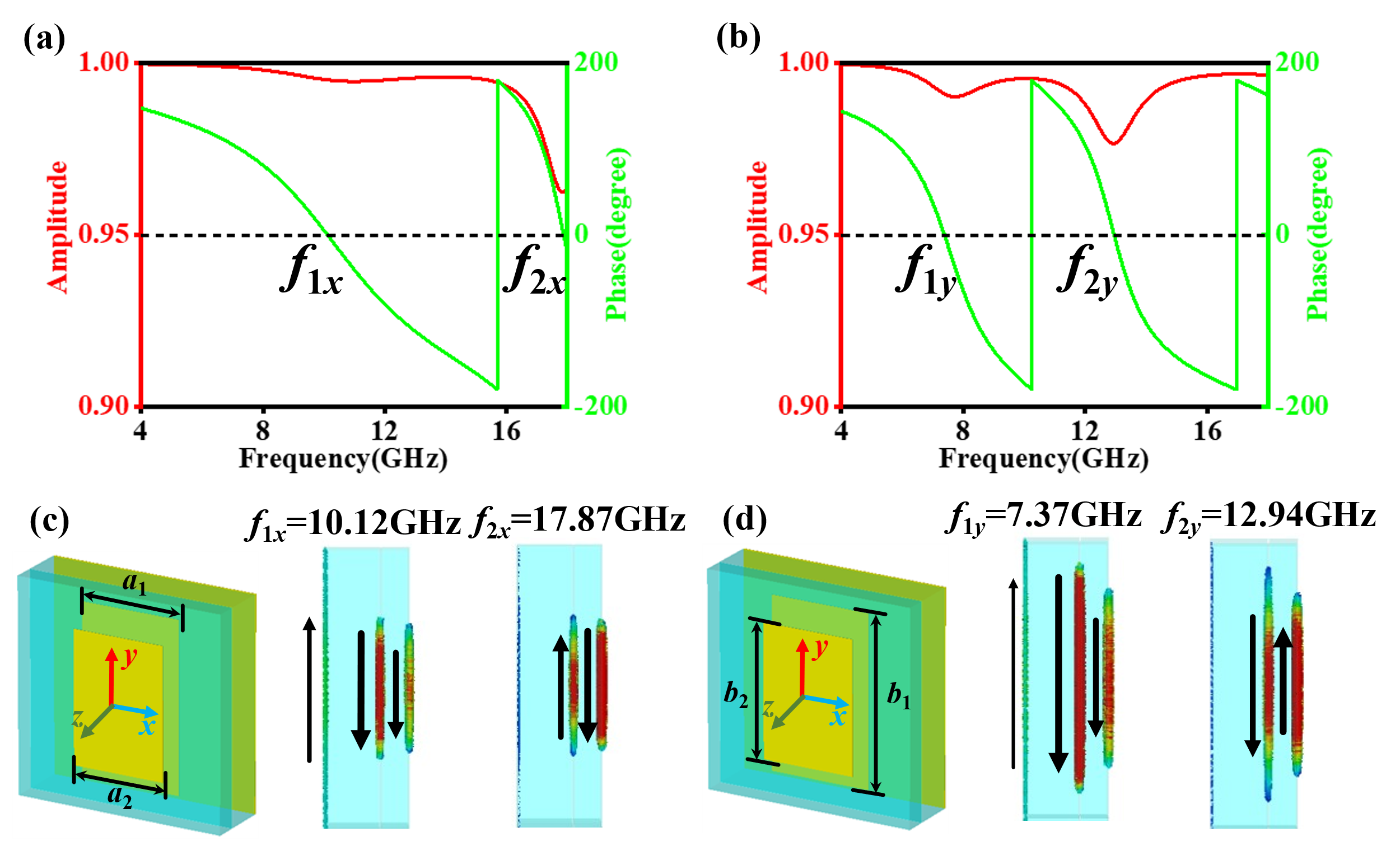


**Figure S1. Schematic of multi-resonant unit cell and the EM response.** The FDTD simulated reflection phase and amplitude for unit cell with *a*_1_=4.8mm, *a*_2_=4.4 mm, *b*_1_=8 mm and *b*_2_=6.2 mm when (a) *x*-polarized waves incident and (b) *y*-polarized waves incident. The schematic of multi-resonant unit cell and the current density distribution along (c) *x*-axis direction in each metallic layer edge at resonant frequency *f*_1_*_x_*=10.12 GHz and *f*_2_*_x_*=17.87 GHz and along (d) *y*-axis direction at resonant frequency *f*_1_*_y_*=7.37 GHz and *f*_2_*_y_*=12.94 GHz.

As already explained in the main text, the proposed unit cell consists of 3 metallic layers separated by two dielectric layers with its schematic shown in Figs. S1(c)-(d). In fact, the parameters of *a*_1_ and *a*_2_ at *x*-axis direction is sensitive to the *x*-polarized waves, while is immune to the *y*-polarized waves. The parameters of *b*_1_ and *b*_2_ is sensitive to the *y*-polarized waves. The corresponding finite-difference-time-domain (FDTD) calculated reflection spectra are shown in Fig. S1(a) when *x*-polarized waves normally incident. The reflection amplitude keeps at a high level of better than 0.95 from 9.5 to 12 GHz. There are two resonances appearing at *f*_1_*_x_*=10.12 GHz, *f*_2_*_x_*=17.87 GHz respectively. According to the current density distribution along *x*-axis direction shown in Fig. S1(c), the magnetic resonance *f*_1_*_x_* is generated by the interaction between the two metallic patch layers and the lower metallic ground while the magnetic resonance *f*_2_*_x_* is due to the interaction between middle metallic patches and the top structure. Fig. S1(b) shows the FDTD calculated reflection spectra when *y*-polarized waves normally incident while the current density distribution along *y*-axis direction is plotted in Fig. S1(d). It is clearly that the magnetic resonance *f*_1_*_y_*=7.37 GHz is generated by the interaction between the two metallic patch layers and the lower metallic ground while the magnetic resonance *f*_2y_=12.94 GHz is due to the interaction between the middle metallic patch and the top metallic layer. We can optimize the structural size to tune the reflection phase spectra via tuning the resonances with the reflection amplitude remaining nearly unchanged for the unit cell.

1. **Principle of phase control for the unit cell of meta-devices**

In this section we analyze the principle of phase control for the unit cell when the unit cell is shined by right-handed circularly polarized (RCP) waves. Fig. S2(a) indicates the reflection phase spectra at high-frequency and low-frequency all could be regulated when changing the parameter *a*_1_. Fig. S2(b) shows the parameter *a*_2_ could regulate the phase spectra at high-frequency. Figs. S2(c)-(d) show that the reflection phase spectra at the low-frequency come regulation with the changing of the parameter *b*_1_ and the reflection phase spectra at the high-frequency comes regulation with the changing of *b*_2_. The analysis can help us to design dual-resonant meta-atoms achieving different reflection phase spectra.


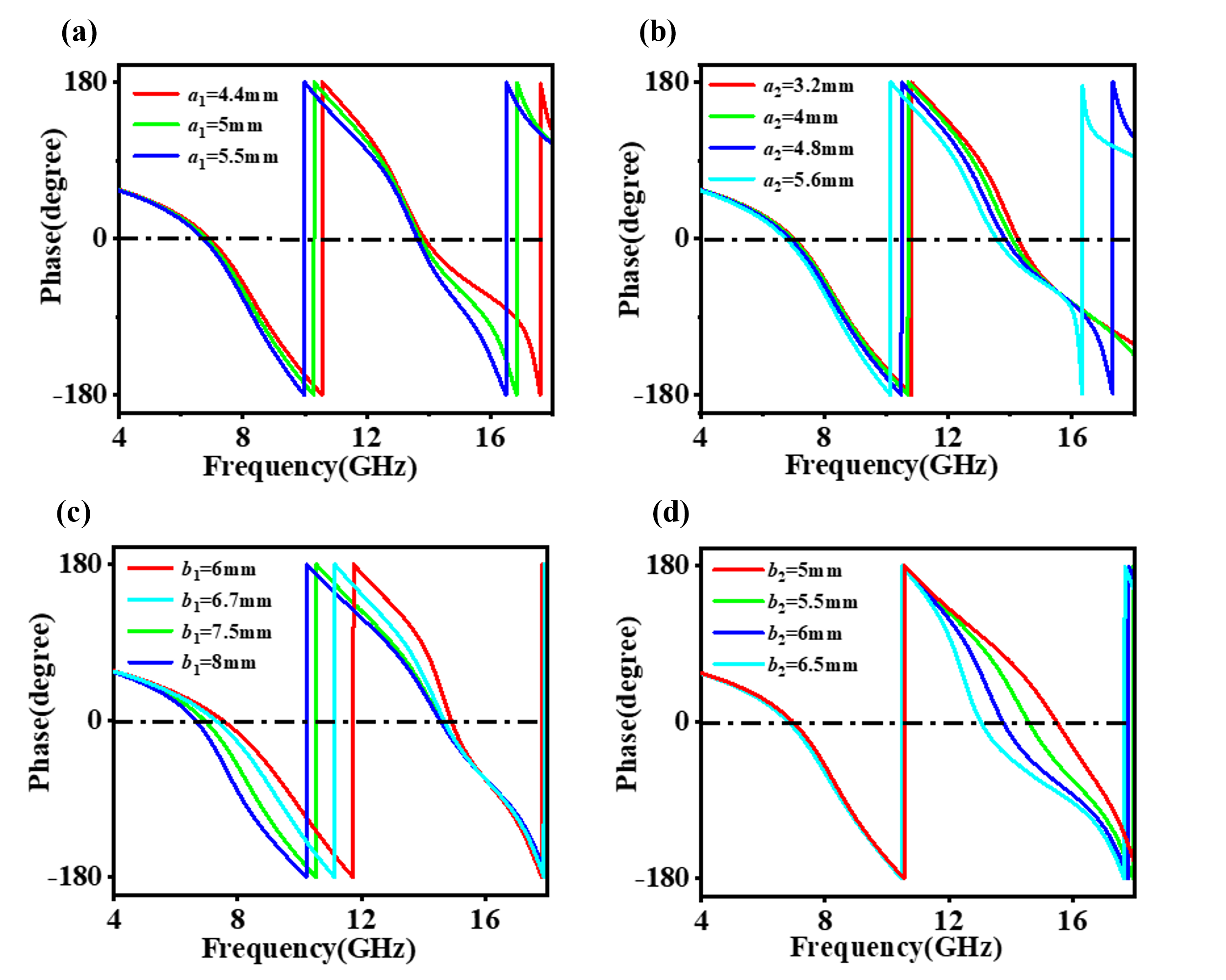


**Figure S2. The variation of the resonant frequency when RCP waves incident.** When *b*_2_=5.9 mm and *b*_1_=7.5 mm, (a) *a*_1_ changes from 4.4 mm to 5.5 mm with *a*_2_=4.8 mm and (b) *a*_2_ changes from 3.2 mm to 5.6 mm with *a*_1_=4.6 mm. When *a*_1_=4.8 mm and *a*_2_=4.4 mm, the variation of the resonant frequency is shown in (c) while *b*_1_ changes from 6 mm to 8 mm with *b*_2_=5.5 mm and (d) *b*_2_ changes from 5 mm to 6.5 mm with *b*_1_=7.5 mm. Inset is the free view of the unit cell.

In the main text, we discuss how to control the dispersion by tuning the structure parameters. Here, we study the variation of reflective phase spectra depending on other parameters as the results shown in Fig. S3. The phase difference *φ_xx_*-*φ_yy_* could maintain around 180° within 9.5 GHz-11.5 GHz when *a*_1_, *b*_1_, *a*_2_, *b*_2_ are adjusted. It also confirms that the unit cell could achieve high-efficiency spin-locked reflection when manipulating the phase dispersion.


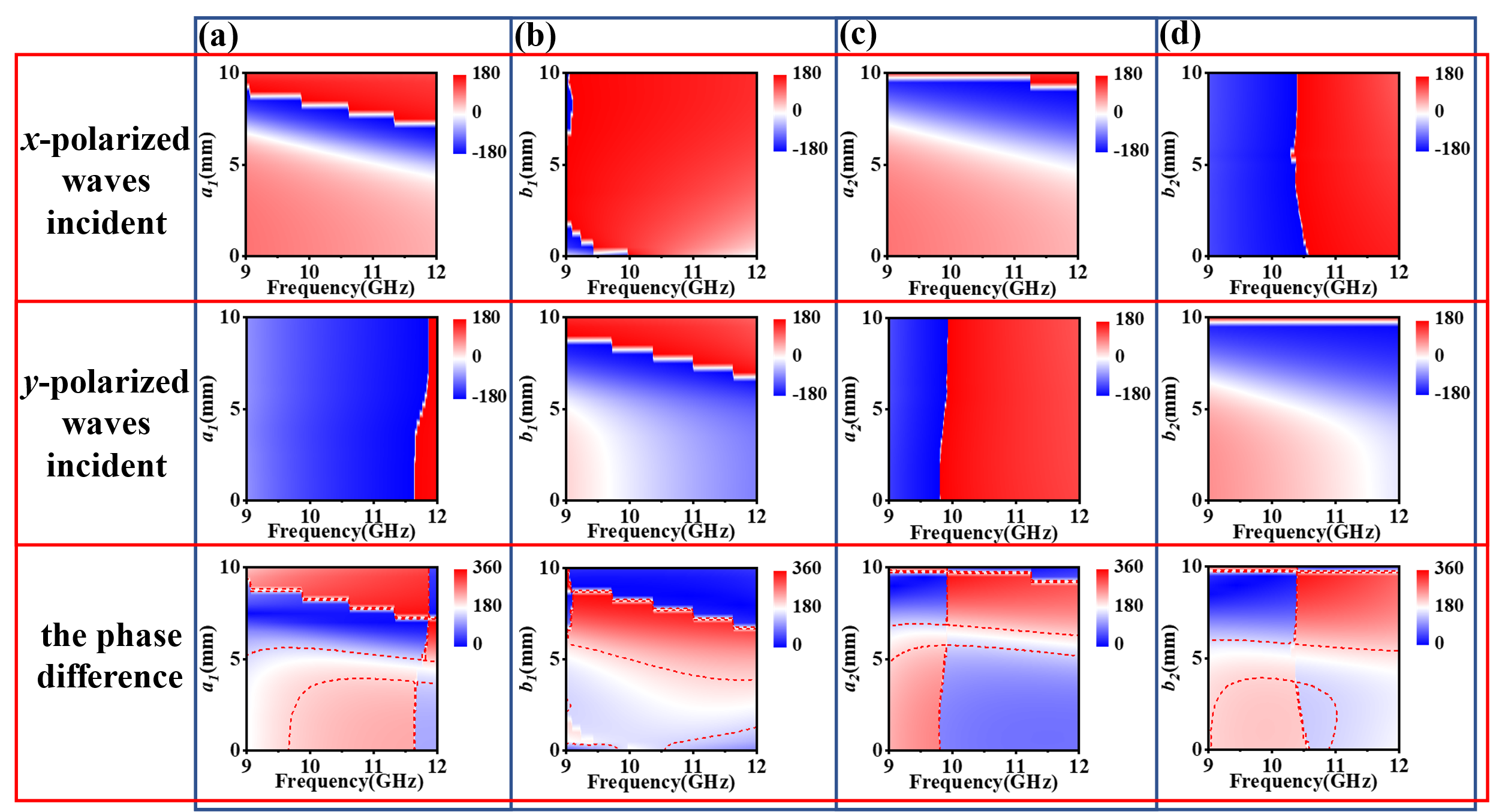


**Figure S3. The phase variation of the integrated-resonant unit elements based on metallic patch.** The phase spectra of *x*-polarized waves incident, *y*-polarized waves incident and the phase difference various frequency and another parameter. For (a), *b*_1_=4 mm, *a*_2_=1 mm, *b*_2_=9 mm. For (b), *a*_1_=9 mm, *a*_2_=5 mm, *b*_2_=6 mm. For (c), *a*_1_=1.5 mm, *b*_1_=8.5 mm, *b*_2_=3 mm. For (d), *a*_1_=8 mm, *b*_1_=4 mm, *a*_2_=5 mm. The phase difference between 180°±30° is outlined by red dotted line.

In the main text, we discuss the strategy to enlarge the phase variation range and to modulate the phase spectra slope by introducing multi-resonant modes. Here, we further analyze the reflection spectra to demonstrate the electromagnetic (EM) manipulation property. At first, we discuss the EM behaviors of the unit when the metal patches are rotated. Fig. S4(a) shows the FDTD calculated reflection spectra of three types of units. It shows that the reflection phase has few changes when only the top patch rotated. When the top and middle metallic patches are rotated together, the variation of phase is twice as that of the rotation angle which matches the Pancharatnam-Berry(PB) principle and the reflection amplitude remaining nearly unchanged for the unit cells.

Then, we analyze the reflection phase of unit 2 (*a*_2_=1 mm) when adjusting *b*_2_, which are shown in Fig. S4(c). It is clear that the high-efficiency reflectivity is very sensitive to the varying of *b*_2_. By increasing the number of patches, as the type 2 and type 3 shown in the insert of Fig. S4(b), the high reflection property can be easily obtained by tuning the structure parameters (see Figs. S4(b), S4(d) and S4(e)).


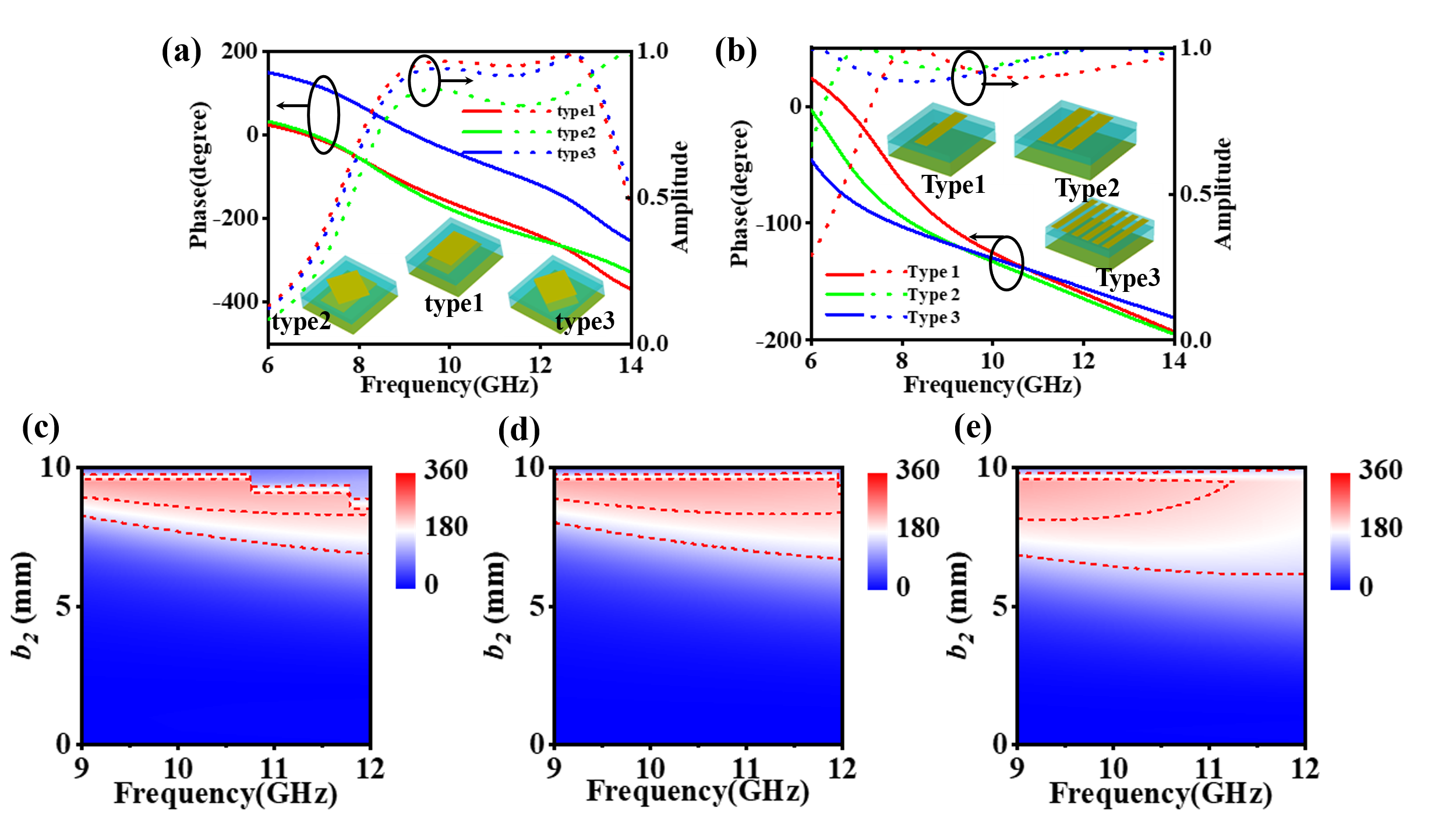


**Figure S4. The reflection spectra of the integrated-resonant unit elements.** (a) Three descendants of unit 3 in main text of Fig. 2(a) with *a*_1_=4.6 mm, *b*_1_=7.5 mm, *a*_2_=4.8 mm, *b*_2_=5.9 mm and the rotation angle set as 60°. (b) The corresponding amplitude and phase spectra of RCP to RCP reflection coefficient for three descendants of unit cell 2 in Fig. 2(a). (c)-(e) The corresponding reflection phase difference of the three types unit cell in (b) when *x*-polarized and *y*-polarized waves incidence respectively.

1. **Detailed design of achromatic deflector**

According to Eq. (1) in the main text, an achromatic meta-mirror should exhibit a linear phase spectrum for each meta-atom while the slope of phase profile is different. Multi-resonant Lorentz model provides guidance to design meta-atom with such phase distribution. We firstly calculated the resonant frequencies of each meta-atom based on the phase maps shown in Fig. 2. Secondly, each meta-atom, composing of different type of composite structures, is optimized carefully. The detailed structure sizes are provided in Fig. S5.


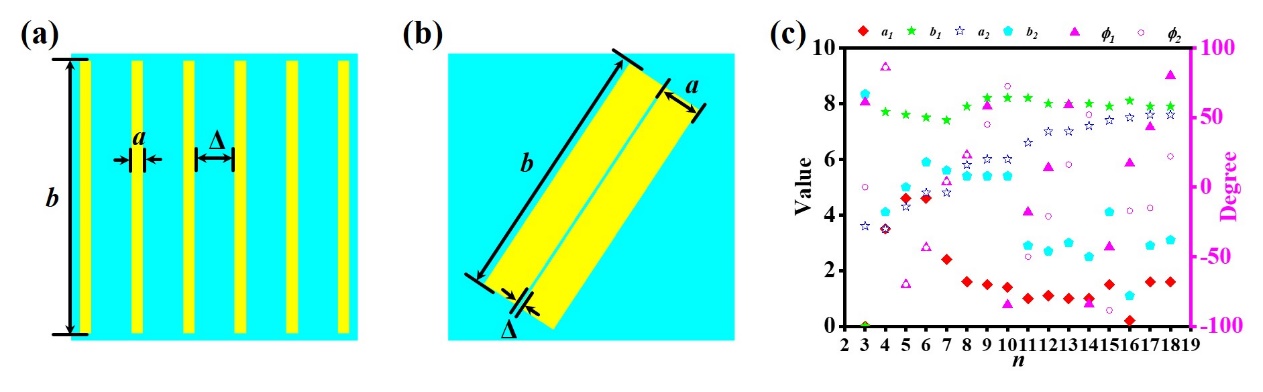


**Figure S5. The special values of the unit cell parameters of the achromatic deflector.** (a) The initial unit cell of the achromatic deflector with *a*=0.4 mm, *b*=9.5 mm, *Δ*=1.8 mm. (b) The second unit cell of the achromatic deflector with *a*=1.4 mm, *b*=9.4 mm, *Δ*=0.1 mm and the patches rotate clockwise 33.5° around the center of unit cell. (c) The special values of parameters from the third unit cell to the eighteenth unit cell of the achromatic deflector. For the rotation angle, the clockwise rotation is positive and the counterclockwise rotation is negative.

1. **Simulation results of scattered-field patterns for achromatic deflector**

In Fig. 3(e) of the main text, we plotted the measured distributions of scattered-field power for our designed achromatic beam deflector. Here, we show in Fig. S6 the corresponding FDTD simulation results, which are found to have well-reproduced all salient features of the experimental results in Fig. 3(e). Both experimental and FDTD results clearly show that the deflection angle doesn’t change as frequency varies, which is quite different from the reported beam deflectors. More importantly, almost all the undesired modes are suppressed, indicating the high-efficiency of our meta-device.


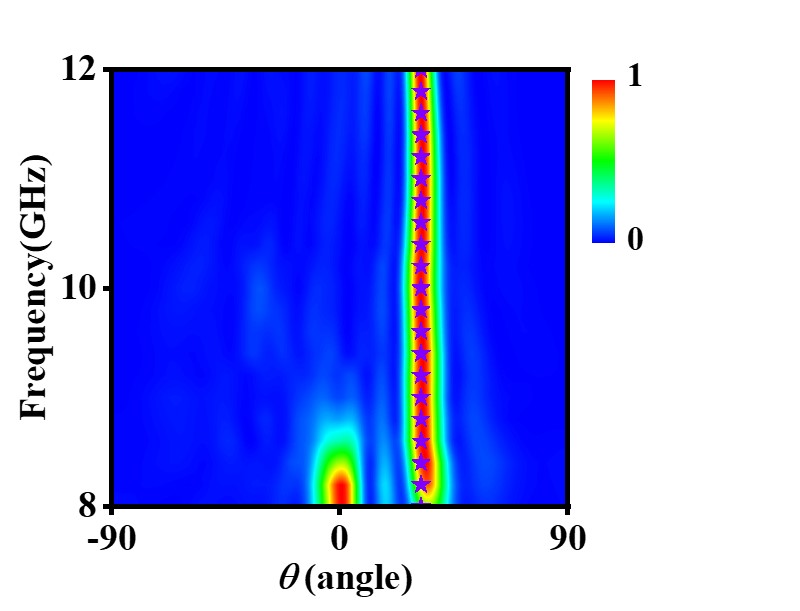


**Figure S6. The far-field FDTD simulation results of achromatic deflector**. For simplicity, there only shows the spectrum of reflective space. The achromatic character is obviously among 9.5 GHz - 11.5 GHz while the anomalous reflection of 32° can be observed.

1. **Detailed design of** **achromatic focusing metasurface**

As stated in the main text, for the unidimensional achromatic focusing metasurface, the phase distribution of the metasurface is calculated based on Eq. (2) and the theoretical phase distributions as a function of element number and frequency are shown in Fig. S7(a). Aided by the two-resonant phase maps (Fig. S2), we can obtain the resonant frequencies of each meta-atom to satisfy the required phase dispersion in Fig. S7(a). Then we tune the parameters of structural size to realize these resonant frequencies. The detailed geometrical parameters (*a*_1_, *a*_2_, *b*_1_, *b*_2_ and *φ*_1_, *φ*_2_) of all meta-atoms involved in our meta-device are shown in Fig. S7(b). What needs illustration is that unit cells in Fig. S7 are numbered from center to edge, with the center unit cell (initial unit cell) numbered 1.


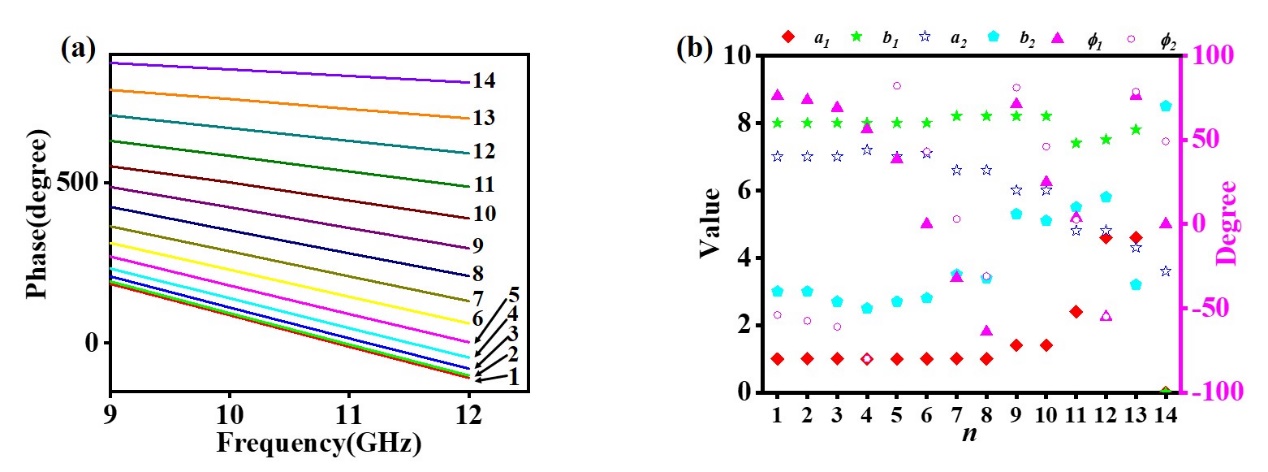


**Figure S7. Design of the achromatic focusing metasurface.** (a) Reflection phase distributions of 14 meta-atoms against frequencies. (b) Distributions of structural parameters *a*_1_, *a*_2_, *a*_3_, *b*_1_, *b*_2_, *b*_3_ and *φ*_1_, *φ*_2_ of the achromatic focusing metasurface studied in Fig. 4 of the main text. The parameters *a*_1_, *a*_2_, *a*_3_, *b*_1_, *b*_2_, *b*_3_ have the unit of mm.

1. **The performance of achromatic focusing metasurface**

The focusing efficiency is experimentally measured in near-field, defined as the ratio between focusing power *P_foc_* and reflected wave power *P_ref_*_2_. The near field experiment is carried in an anechoic chamber while the transmitter is a standard circularly-polarized horn which could emit RCP plane waves propagating along -*z*-axis direction. Meanwhile, the reflected EM waves keep the same charity as RCP.

There are indeed different components at the local point, such as ***E_x_***, ***E_y_*** and ***E_z_*** fields. We measured the ***E_x_*** and ***E_y_*** components of reflective RCP waves respectively to extract the efficiency of focusing beam. Fig. S8 plots the simulated and measured *x*-polarized electric field intension (|***E_x_***|^2^) distribution at *xoz* plane at 9.5 GHz, 10.5 GHz, and 11.5 GHz respectively. The integration of power along the *x*-axis direction is defined as reflecting power *P_ref_*_2_*_x_* while the integration of power at concentration part (the gray area in Fig. S8) is defined as focusing power *P_focx_*. Then we can obtain that focusing efficiency as *P_focx_* /*P_ref_*_2_*_x_*. The similar method is used to evaluate the focusing efficiency for the *y*-polarized electric field. It is interesting that the focusing efficiency for the *x* polarization and *y* polarization is consistent. We can infer that the *z* component exhibits the consistent focusing efficiency. Therefore, we calculate the focusing efficiency as:

$\eta_{2}\text{=}\frac{P_{foc}}{P_{ref2}}=\frac{1}{2}(\frac{P_{focx}}{P_{ref2x}}+\frac{P_{focy}}{P_{ref2y}})$. (S1)


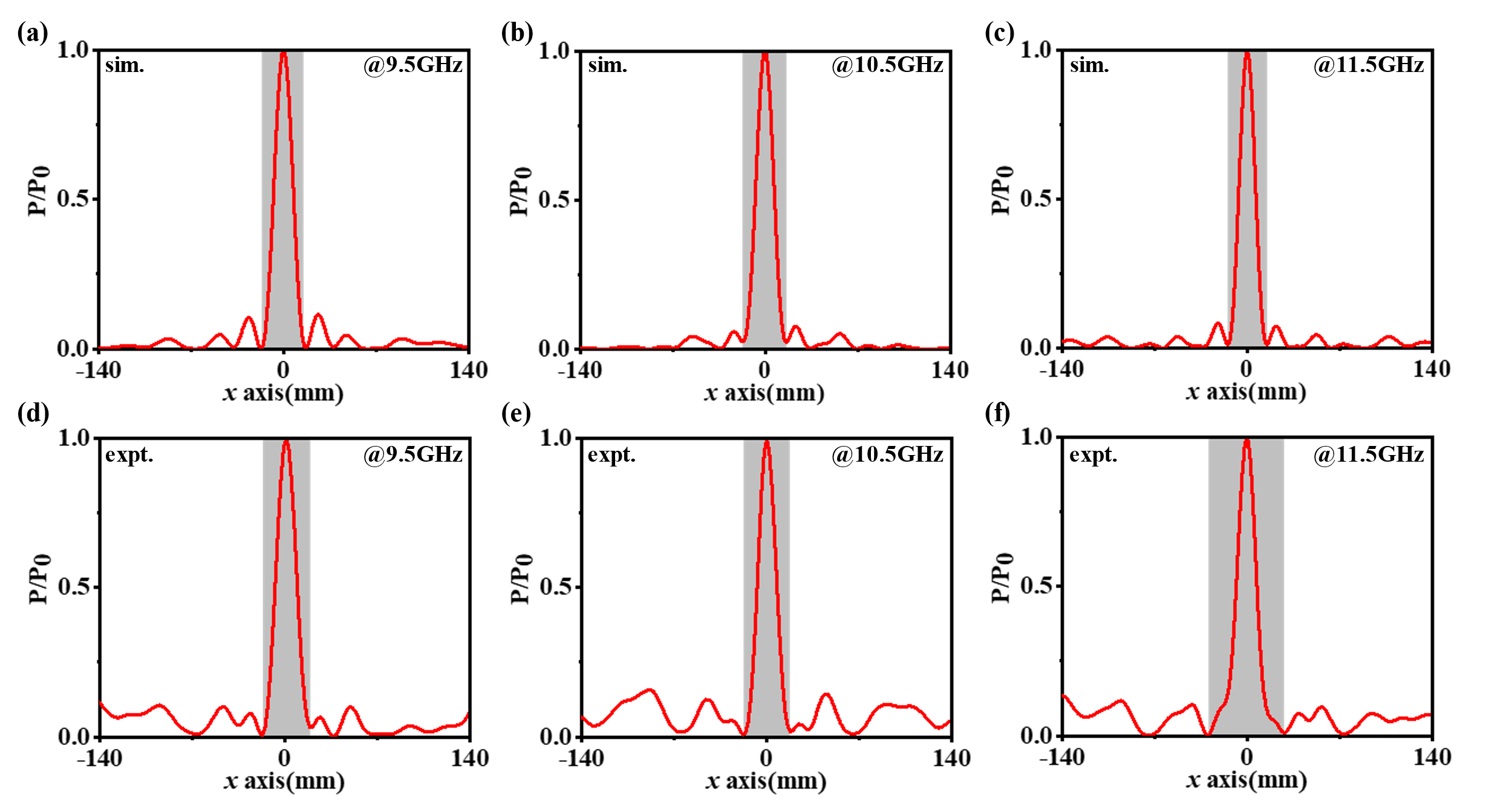


**Figure S8.** **The normalized energy distribution along *x*-axis direction on horizontal section plane (*xoz* plane, *y*= 0 mm) for *x*-polarized electric field component.** The simulation results at (a) 9.5 GHz, (b) 10.5 GHz, (c) 11.5 GHz and the experiment results at (d) 9.5 GHz, (e) 10.5 GHz, (f) 11.5 GHz.

The experimentally measured *x*-polarized electric field component distributions of our designed achromatic focusing metasurface are plotted in Fig. 5(f)-(h) in the main text when the sample is illuminated by normally incident RCP EM waves. Here, we plot the measured *y*-polarized electric field component distributions on *xoz* plane (*y*=0 mm) at 9.5 GHz, 10.5 GHz, 11.5 GHz in Fig. S9(a)-(c) when RCP EM waves are normally incident. We can see clearly that most of the reflective electric fields are focused to the center of the plane and the achromatic focusing performance is obviously, which are consistent with the distribution for the *x*-polarized component.


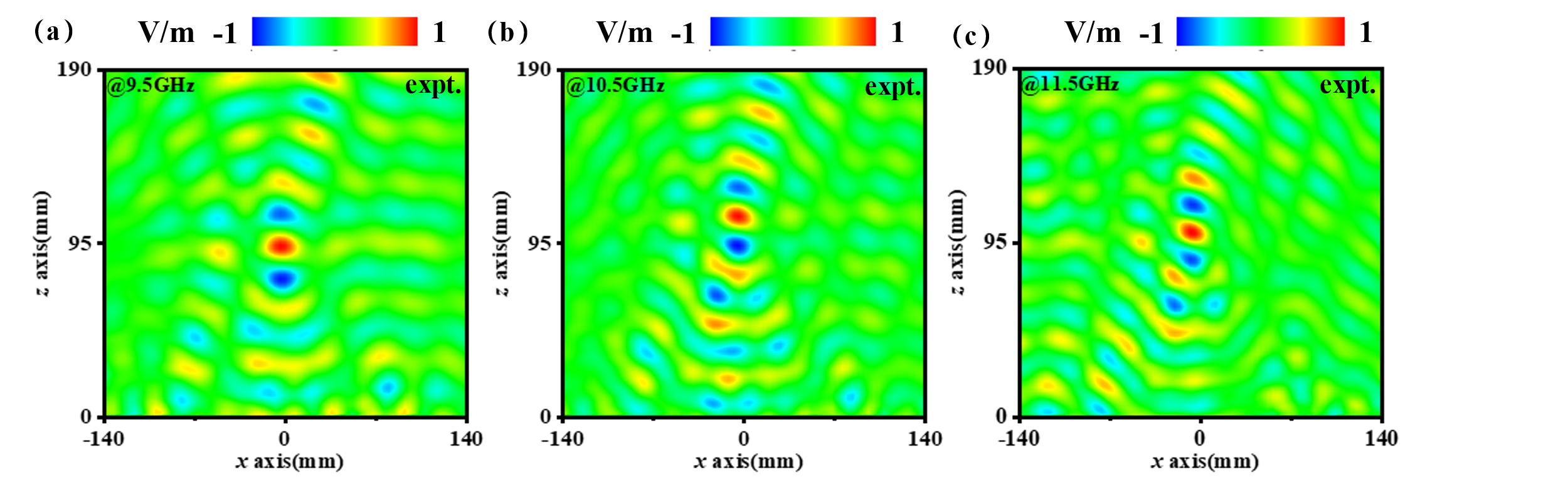


**Figure S9. Experiment results of near-field electric field distribution** **for y-polarization on *xoz* plane (*y*=0 mm) at (a) 9.5 GHz, (b) 10.5 GHz, (c) 11.5 GHz.**

1. **Measurement setup for the designed meta-devices**


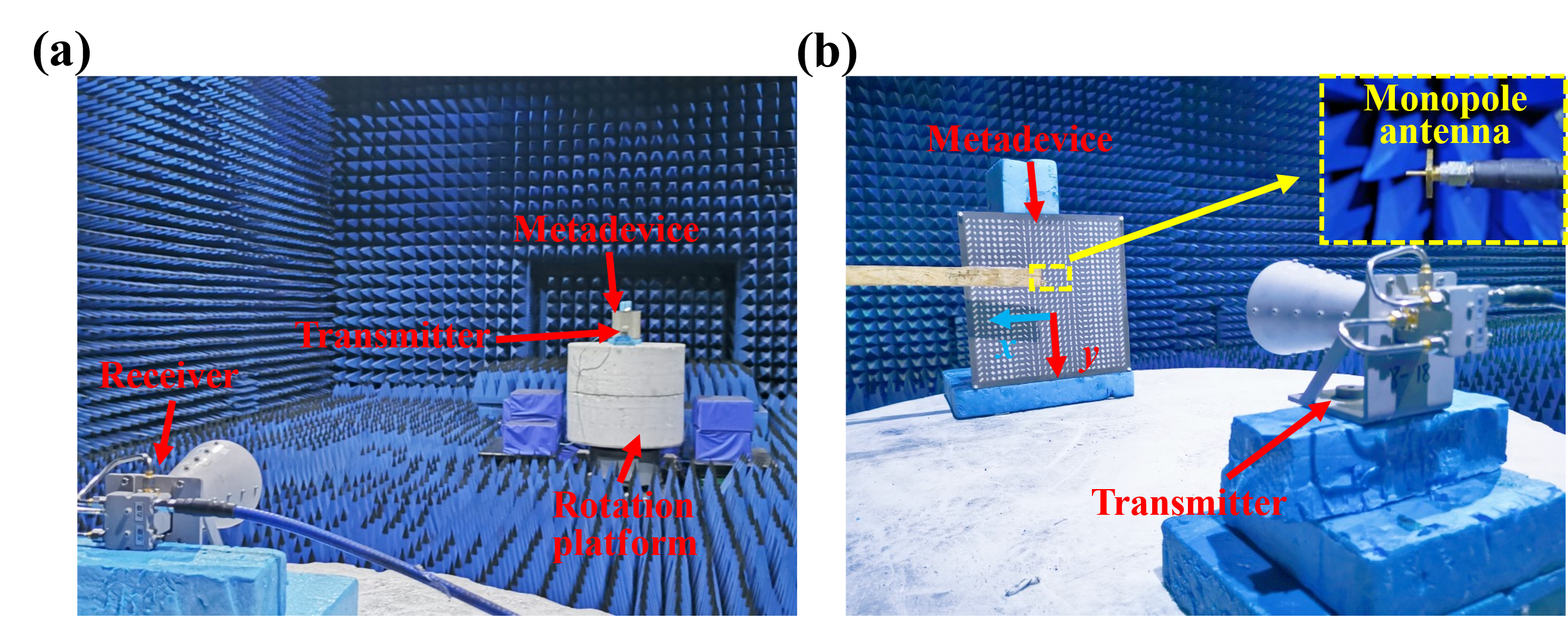


**Figure S10.** **Schematics of the experimental setup of our designed meta-device.** (a) Experimental setup to measure far-field scattering patterns of the meta-devices. (b) Experimental setup to measure ***E*** distributions at the frequency band of 9.5-11.5 GHz as the meta-device is illuminated by normally incident RCP waves.
